# Estimating dengue vector abundance in the wet and dry season: implications for targeted vector control in urban and peri-urban Asia

**DOI:** 10.1179/2047773212Y.0000000063

**Published:** 2012-12

**Authors:** Khin Thet Wai, Natarajan Arunachalam, Susilowati Tana, Fe Espino, Pattamaporn Kittayapong, W Abeyewickreme, Dilini Hapangama, Brij Kishore Tyagi, Pe Than Htun, Surachart Koyadun, Axel Kroeger, Johannes Sommerfeld, Max Petzold

**Affiliations:** 1 Department of Medical Research (Lower Myanmar), Yangon, Myanmar; 2 Centre for Research in Medical Entomology, Indian Council of Medical Research, Madurai, India; 3 Center for Health Policy and Social Change, Yogyakarta, Indonesia; 4 Research Institute for Tropical Medicine, Alabang, Muntinlupa City, Philippines; 5 Center of Excellence for Vectors and Vector-Borne Diseases, Faculty of Science, Mahidol University at Salaya, Nakhon Pathom, Thailand; 6 Department of Parasitology and Molecular Medicine Unit, University of Kelaniya, Sri Lanka; 7 Department of Disease Control, Ministry of Public Health, Nonthaburi, Thailand; 8 Special Programme for Research and Training in Tropical Diseases (TDR), World Health Organization (WHO), Geneva, Switzerland; 9 Liverpool School of Tropical Medicine, Liverpool, UK; 10 Centre of Applied Biostatistics, The Sahlgrenska Academy, University of Gothenburg, Gothenburg, Sweden

**Keywords:** dengue vectors, vector ecology, eco-health, vector breeding, weather dependence, targeted vector control, productive container types

## Abstract

**Background:**

Research has shown that the classical Stegomyia indices (or “larval indices”) of the dengue vector *Aedes aegypti* reflect the absence or presence of the vector but do not provide accurate measures of adult mosquito density. In contrast, pupal indices as collected in pupal productivity surveys are a much better proxy indicator for adult vector abundance. However, it is unknown when it is most optimal to conduct pupal productivity surveys, in the wet or in the dry season or in both, to inform control services about the most productive water container types and if this pattern varies among different ecological settings.

**Methods:**

A multi-country study in randomly selected twelve to twenty urban and peri-urban neighborhoods (“clusters”) of six Asian countries, in which all water holding containers were examined for larvae and pupae of *Aedes aegypti* during the dry season and the wet season and their productivity was characterized by water container types. In addition, meteorological data and information on reported dengue cases were collected.

**Findings:**

The study reconfirmed the association between rainfall and dengue cases (“dengue season”) and underlined the importance of determining through pupal productivity surveys the “most productive containers types”, responsible for the majority (>70%) of adult dengue vectors. The variety of productive container types was greater during the wet than during the dry season, but included practically all container types productive in the dry season. Container types producing pupae were usually different from those infested by larvae indicating that containers with larval infestations do not necessarily foster pupal development and thus the production of adult *Aedes* mosquitoes.

**Conclusion:**

Pupal productivity surveys conducted during the wet season will identify almost all of the most productive container types for both the dry and wet seasons and will therefore facilitate cost-effective targeted interventions.

## Introduction

Dengue is the fastest re-emerging arboviral disease worldwide imposing a heavy economic and health burden on affected individuals, their families and thereby the health system as a whole.^1^–^3^ In the absence of a specific drug for treatment and an effective vaccine available for public health use, vector control measures to reduce mosquito densities and proper case management to minimize case fatality^4^ are the presently available best strategic options. However, large dengue outbreaks continue to occur annually with the disease extending to new geographical areas.^1^ Nathan *et al*. (2006)^5^ have stated that routine interventions against the immature stages of the vector often have little effect,^2^ while vertical interventions are often short lived.^1^ Therefore, it is appropriate at this juncture, to study whether the targeted management of the most mosquito-productive containers is more effective than mass efforts to eliminate all potential breeding places in different socio-ecological settings.

A number of practical dengue vector control tools and approaches, often suitable for specific container types, are now available including targeted interventions in the container types producing more than 70% of *Aedes* pupae,^4^ biological control measures^4^,^6^ and some conventional as well as innovative insecticidal applications.^4^,^7^,^8^,^32^ These are sometimes most effectively deployed within integrated community-based vector management efforts,^9^ and efficacy can often be optimized by targeting the most productive container types.^10^,^11^ This approach can only be implemented after identifying productive container types through the calculation of pupal indices (e.g. PPI, Pupae per Person Index), which are determined by the data collected in pupal productivity surveys.^12^ The application of subsequent control strategies should be based on the knowledge of site-specific contextual factors related to the ecosystem^14^ and the social and political setting^15^,^16^ including municipal and governmental services, in addition to knowledge on local vector ecology. Such integrated efforts have been labeled the ‘eco-health approach’ to dengue vector control^15^ and fit into an integrated vector management (IVM) framework.^9^,^17^

The present study is part of a multi-site research programme in six Asian countries which aimed at elucidating contextual factors related to dengue vector abundance in a comprehensive way and then to design and implement site-specific interventions. The programme was guided by a conceptual framework which included ecological, biological (entomological) and social (‘eco-bio-social’) determinants of vector density as key factors for dengue transmission (see details of the comparative situation analysis in Arunachalam *et al.* 2010).^18^

The primary objective of this study was to analyze vector breeding patterns in six different country settings, particularly their preference for specific container types during the dry and wet season, in order to re-assess the concept of “productive containers” (as opposed to simply larval infestation) for vector control purposes and to identify the optimal timing for determining *Aedes* production. In this paper, we report on findings from the situation assessment, particularly variables related to dengue pupal vector abundance in the dry and in the wet season, in order to provide data on the seasonal variation of habitats of the immature stages and the optimal timing for conducting pupal productivity surveys. The total number of *Aedes* pupae^11^ encountered in a neighborhood (or study cluster) is used as a proxy indicator for adult dengue vector density, as roughly 80% of pupae develop to adult mosquitoes;^12^ the “Pupae per Person Index” (PPI) is used as an indicator for the ratio between vector abundance and human population to assess dengue transmission risk.

## Study Sites and Methods

### 1. Study sites and timeline

Table 1 presents a synopsis of the six study sites in Asia including larger cities in India (Chennai), Indonesia (Yogyakarta), Myanmar (Yangon) and the Philippines (Mutinlupa City) as well as middle-sized provincial towns and their surrounding peri-urban areas in Sri Lanka (Gampaha district) and Thailand (Chachoengsao Province). The generally middle class study neighborhoods included mainly well-developed urban areas with good to acceptable public services (electricity, regular piped water supply, waste disposal through public services, paved streets) with some exceptions in Myanmar (water mainly from wells); Sri Lankan (waste collection in a minority of study neighborhoods) and Philippines (large proportion of lower-class residents). The field studies were conducted from mid-2007 to mid-2008 and the data were analyzed from 2009 to 2011.

**Table 1:** Short description of study areas

Country	Study location and climate	Ecological characteristics
India	•Chennai city, TamilNaduState, 4.55 million population	The study clustersincluded well developed urban areas with electricity and paved streets (90%), generally piped water and indoor toilets, and waste collection at least once per week. 70% of the clusters consist of residential areas, predominantly middle class with good/satisfactory housing. Often these are two to five storey buildings with patios or gardens. Almost half of neighborhoods have market places, most of them with schools, and/or cemeteries. There were relatively few green areas with or without leisure activities. Visible garbage dumps and open water pools were found in one third of study clusters and tire capping facilities in one quarter.
	•Annual average temperature of 31.2°C, average rainfall of 1245.7 mm, relative humidity = 62%–86%.	
Indonesia	•Yogyakarta city, within Yogyakarta province, 435,236 inhabitants	The study clusters included well developed urban areas with electricity and paved streets. Households had mostly indoor toilets but water was mainly drawn by hand from wells. Waste collection was conducted at least once per week in all study neighborhoods. Study site was half residential, half mixed commercial/residential areas of the middle class (no lower class strata included) with good/satisfactory housing conditions. Only one storey buildings, generally with a patio/garden and bushes or trees. Half of study neighborhoods with green areas, with or without leisure activities, almost half of them had schools, quite often cemeteries but rarely market places. Tire capping facilities were found in all study clusters and visible garbage dumps in half of them but no open water pools.
	•Average annual temperature of 27.8°C and average rainfall 2,156 mm, humidity = 72%–87%.	
Myanmar	•YangonCity, Yangon Division, 4.8 million inhabitants	The study clusters were overall reasonably well developed urban areas with electricity and many (75%) with paved streets; water was mainly drawn by hand pumps (79.1% of households); most toilets (83.1%) were in the patio - half of them latrines, half septic tanks. Waste collection at least once per week in all study neighborhoods. Three quarter of neighborhoods were mainly residential, the remainder mixed commercial/residential areas. In the majority the poorer social strata were included in the study, but the housing conditions, mainly one storey buildings, were generally satisfactory to good. One third had patios/gardens and some of these trees and/or bushes. Green areas were frequent but rarely for leisure activities. There were no neighborhoods with cemeteries, half of them with schools and almost half of them with small market places. Many study clusters had visible garbage dumps or open water pools and about one third had tire capping facilities.
	•Average annual temperature of 24°C average annual rainfall for 2007 was 295 mm, humidity = 67%–91.9%.	
Philippines	•MuntinlupaCity, south of the Manila metropolitan area, 446,830 inhabitants.	Overall well developed urban areas with electricity and paved streets, water was generally drawn by hand pumps but most houses had indoor toilets. Waste collection at least once per week in 83.3% of study clusters. Only residential areas, half of them with middle class the other half with lower class residents, all houses being of a good or satisfactory quality. Half of the houses were one storey buildings the other half two or more storey buildings; one third had patios or gardens and one quarter has trees or bushes. All clusters had green areas, half of them for leisure activities; all had schools. Almost half of the study clusters had market places but there were no cemeteries included. Visible water pools were frequent (half of the study clusters) and visible garbage dumps in one third of clusters; tire capping facilities were seen in one quarter of the neighborhoods.
	•Average annual temperature for 2006 was 31°C, average annual rainfall was 186 mm, averagerelative humidity = 77%.	
Sri Lanka	•Gampaha district, Western Province bordering district of Colombo, 2.1 million inhabitants	Gampaha district is a rapidly urbanizing district in close proximity to Colombo. Study clusters include well developed urban and peri-urban areas with electricity and frequently paved streets. Water was either drawn by hand pumps (56.4%) or – in town centers - was piped (43.4%) but most houses had outdoor toilets. Waste collection was only done at least once per week in 40% of study neighborhoods. Most clusters had a mix of commercial and residential premises and also a mix of middle and lower class residents; however, 95% of houses –all of them with mainly one storey buildings- were reported to be satisfactory to good. The large majority had patios or gardens and most of them trees or bushes. Most neighborhoods had green areas and several of them for leisure activities. Market places, cemeteries and schools were relatively rare in the study areas. Visible garbage dumps were frequent but open water pools relatively rare. Only 15% of study clusters had tire capping facilities.
	•Average annual temperature is 27.8°C, annual rainfall > 2500 mm, humidity = 70%–90%.	
Thailand	•ChachoengsaoProvince, 120 km east of Bangkok; 654,206 inhabitants.	Overall well developed town areas with electricity and paved streets, generally piped water and indoor toilets. Waste collection at least once per week in all study clusters. Mainly mixed residential/commercial areas predominantly with a mix of middle and lower class residents but in general with good/satisfactory housing, mainly with two to five storey buildings with patios or gardens (69%) and often with bushes and trees (54.8%). There were few market places, few schools, no cemeteries but a number of green areas (66.6%), many of them for leisure activities. There were only few visible garbage dumps and open water pools and no tire capping facilities at all.
	•Average temperature is 27.96°C, the average annual rainfall = 1284.06 mm and the average humidity = 76%.	

### 2. Sampling approach

All study sites followed a joint protocol of using 20 (India, Myanmar, Sri Lanka) or 12 clusters (Indonesia, Philippines, Thailand) for conducting the household surveys, cluster background surveys and entomological surveys (see below). A cluster was defined as a neighborhood of around 100 houses with public (non-residential) areas between or around the houses.

#### Sample size

The sample size was calculated based on the intervention studies expected for Phase II of this research program. It was based on a post-intervention cross-sectional testing of pupae per person between the intervention and control clusters using a two-level hierarchical model with clustering at study cluster level. Sample size reflected a desired significance level of 5% and a power of 80%. Further, mean levels of pupae per person in control and intervention areas were assumed to be 3.0 and 0.3, respectively, based on previous studies.^19^ For a negative binomial distribution with a dispersion coefficient of 0.02 and an intra-cluster coefficient of 0.05 the required number of clusters was 8.9 per study arm when sampling 100 households per cluster. The sampling was then increased to 10 clusters per arm per site, for a total of 20 clusters per site in 3 countries and (for operational reasons) kept at 6 clusters per arm in 3 countries. The assumption of a negative binomial distribution was aimed to account for potential over-dispersion of data, i.e. if obtaining a large number of zero counts in combination with some extremely high counts, which sometimes occurs in these kinds of studies.

#### Grid sampling of study clusters

The selection of study clusters was based on the methodology describes by *Troyo et al.*.^13^ A map of each study site was generated using Google Earth software (Google Inc., Mountain View, CA, United States of America). A grid with 200 squares was overlaid on the map, and the squares were numbered. 20 squares were randomly selected using a simple random number generator, with the exception of the sites in Indonesia, Philippines and Thailand where, due to operational difficulties, only 12 squares were selected.

#### Definition of clusters within squares

In each of the selected squares, the left lower corner was identified on the map and the exact location was determined using a GPS and its physical location was found in the actual city. Starting from this point, the closest crossing of two streets was identified, one street representing the vertical line of the square in the map and the other the horizontal line of the square. Then, the researchers went roughly 100 meters along the horizontal street, turned left and looked into the ‘vertical’ direction and identified a street that was parallel to the first horizontal street, obtaining a U-shaped form. In order to close the U to define the cluster area, the researcher looked for 100 premises (houses, flats, small business units) within the U shaped area. After arriving at a total of 100, the U was closed providing the final border of the cluster. A simple map was drawn for orientation. If the square fell over a football ground or large park or any open public space, then the next closest corner of two crossing streets was used to construct the U. All premises as well as public and private open spaces were included in the cluster analysis.

### 3. Surveys

#### Household survey

For characterizing the study population and assessing knowledge, attitudes and practices related to dengue vector control, a household survey was conducted during the wet season in all study sites by trained field workers, using the same questionnaire.^18^

#### Entomological survey

Surveys to assess container infestations with immature mosquito stages were conducted during the dry and the wet season according to standard operating procedures^20^ by two-to-six university or full-time vector control staff who were re-trained in the survey procedures and use of the common data collection instrument. Household areas including intra-domestic and peri-domestic spaces as well as public (non-household) spaces in each cluster were inspected. Only containers with water (“wet containers”) were examined. The containers were classified according to type, source of water, volume, location, presence of vegetation, presence of larval control measures and presence of a proper/suitable cover. For larvae, the surveyor determined the presence or absence of *Aedes* larvae in each container. For pupae, the surveyor counted all the pupae present in each container. A 10% sample was taken back to the laboratory for species confirmation. In some sites where large water containers (Myanmar) or wells (Indonesia and less in India) were encountered, the sweeping method^20^,^21^ or the funnel technique was employed.^12^,^22^ In the Philippines, a correction factor was applied in large water containers (>200L) for improving the estimate of total pupal counts.^23^ During the dry and wet season a sample of pupae from different container types was examined in the laboratory and left to develop into adults. The adults were then identified by species and sex. Between 90% to 100% of samples were *Ae. aegypti* with a small number of *Aedes albopictus* in Sri Lanka, Philippines and Thailand. As such, in this paper we will report all larvae and pupae encountered as immature stages of dengue vectors.

### 4. Data management and analysis

All data were double checked by field supervisors before entry into the database. Double entry for quality assurance was done by trained data entry personnel. All data files were checked and cleaned by data entry supervisors. EpiData 2.0 (http://www.epidata.dk) was used as the data entry and management software since it is equipped with range check and skip check, as well as data export capability. The data files from all study sites were merged and analyzed jointly in the data management centre at Gothenburg, Sweden. Analyses regarding factors associated with pupal production were performed for different units of analysis: container (pupae counts, pupae/larvae positivity), household (pupae counts) and study cluster (pupae per person, house index, Breteau index, pupae per hectare). For container-level analysis, clustering of observations at study cluster level was assumed and two-level hierarchical models were used for estimation. Count data were analyzed using negative binomial regression. Covariates were included in the regression models based on assumed dependencies. STATA version 11.1 was used in the analysis.

### 5. Meteorological data and information on reported dengue cases

Monthly averages of temperature and relative humidity were collected from local meteorological stations for the five years preceding the study (in order to identify general patterns). Information about reported dengue cases was obtained from the passive surveillance system of Ministries of Health for the same period.

## Results

### Study populations

In the six study sites a total of 9,391 households with 42,361 inhabitants were visited and interviewed (Table 2). Across all sites, interviewees were mostly (88.9%) older than 25 years of age and to a large extent (65.7%) females. The number of people per household varied from 5.2 persons per household in Yangon (Myanmar) and 4.9 in Mutinlupa City (Philippines) to fewer in the peri-urban study site in Thailand with 3.4 persons per household.

**Table 2:** Overview of container infestation measures in the wet and dry seasons (Standard Deviation in brackets)

	Season	India	Indonesia	Myanmar	Philippines*	Sri Lanka	Thailand	Total (or) Average
**No. of clusters**	Dry	20	12	20	6	20	12	90
	Wet	20	12	20	12	20	12	96
**No. of water containers per cluster**	Dry	435	401	880	314	138	598	461.00
	Wet	543	488	948	223	157	693	508.67
**No. of pupae per cluster**	Dry	34	127	246	73	4	11	82.50
	Wet	96	205	253	157	28	46	130.83
**BI**	Dry	8.8 (±5.20)	26.1 (±12.6)	49.0 (±19.7)	12.6	6.6 (±3.78)	25.9 (±25.8)	21.50 (±20.7)
	Wet	28.1 (±18.1)	57.4 (±21.8)	66.1 (±18.3)	23.7 (±22.7)	11.4 (±6.79)	48.9 (±18.8)	39.27 (±26.9)
**HI**	Dry	7.2 (±3.71)	20.1 (±9.88)	27.5 (±6.68)	9.9	5.4 (±2.78)	18.9 (±23.1)	14.83 (±14.41)
	Wet	19.6 (±12.2)	34.1 (±11.5)	36.2 (±7.17)	16.4 (±11.6)	9.4 (±5.60)	29.9 (±8.91)	24.27 (±13.87)
**CI**	Dry	2.2 (±1.14)	6.1 (±2.38)	5.7 (±2.10)	3.6	4.8 (±2.49)	5.0 (±5.34)	4.57 (±3.31)
	Wet	5.7 (±2.78)	11.1 (±4.16)	7.1 (±1.41)	12.5 (±9.77)	7.1 (±3.69)	8.5 (±3.85)	8.67 (±4.97)
**PPI (Pupae per person)**	Dry	0.073 (±0.090)	0.296 (±0.360)	0.461 (±0.281)	0.157	0.008 (±0.011)	0.021 (±0.042)	0.17 (±0.26)
	Wet	0.207 (±0.191)	0.446 (±0.190)	0.47 (±0.197)	0.288 (±0.202)	0.070 (±0.090)	0.129 (±0.097)	0.27 (±0.23)
**Total number of pupae per site**	Dry	680	1524	4920	438	80	132	7774
	Wet	1920	2460	5060	1884	560	552	12436

*Operational difficulties limited the number of study clusters in the Philippines during the dry season and no standard deviation was estimated.

### Rainfall and vector abundance

**Figure 1:**
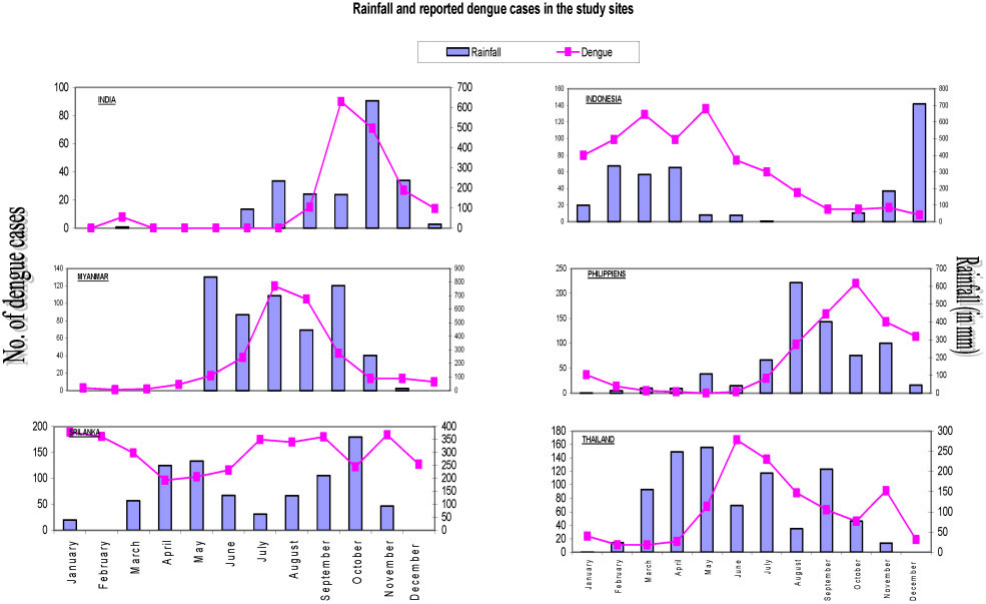
The association of rainfall and reported dengue cases over 5 years in the 6 study sites.

The relationship between average monthly rainfall and reported dengue cases is illustrated in Figure 1 confirming the popular wisdom of a ‘dengue season’ in each site; only the Gampaha district in Sri Lanka had a bimodal rainfall pattern with dengue transmission at high levels year-round. Weekly or monthly temperature was not included in our analysis.

#### Vector ecology with a focus on immature stages

We analyzed, using pupal-demographic surveys (see methods) which of the water containers in the different study sites, both in the domestic environment and in public spaces, were the most frequent *Aedes* breeding places in the dry and the rainy seasons; and which were the most productive for pupal development (Table 2). The specific findings are detailed in the following sections.

##### Water containers and vector breeding in the dry and wet season

**Figure 2:**
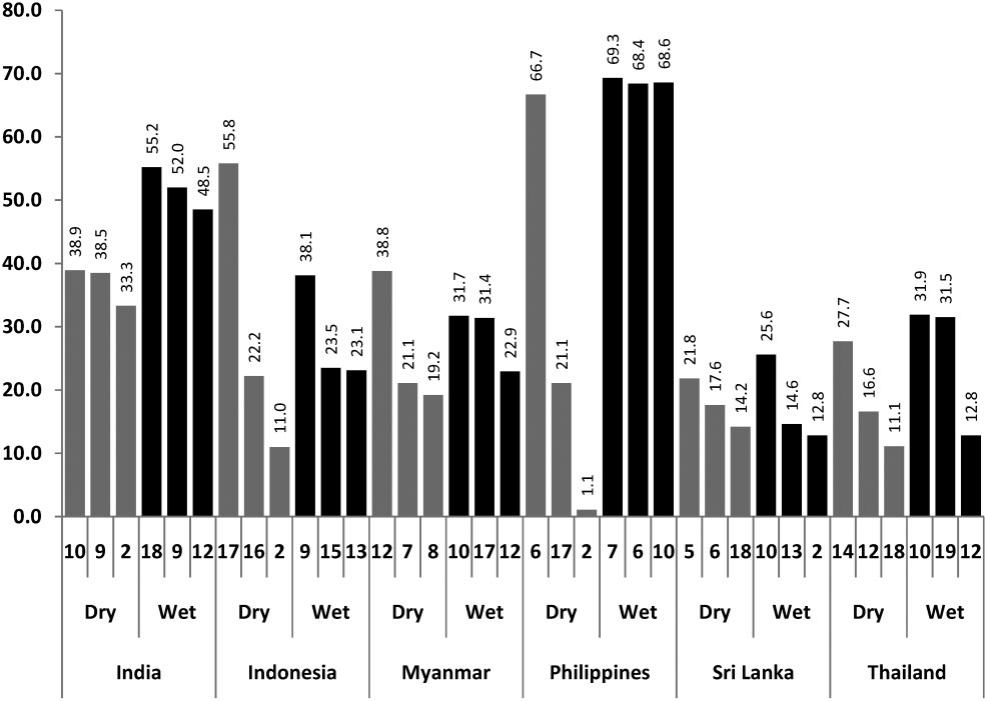
Stegomyia index (CI): The 3 most frequently positive container types for any immature *Aedes* stage (% containers with larval and/or pupal infestation; Code of container type at the bottom of each column). Container codes: 2 cement tank; 5 metal pot; 6 earthern (clay) pots; 7 ceramic jar; 8 bowl; 9 flower vase; 10. tire; 12 discarded containers (tins, bottles etc); 13 natural containers (coco nut shells, plant axilli etc.); 15 bucket; 17 spiritual flower bowl; 18 grinding stone.

There was on average an increase of water holding containers from the dry to the wet season of 8.8% (from 477 to 519 containers per study cluster; Table 2). Such an increase happened across all sites with the exception of the Philippines where people stored more water during the dry season. Figure 2 summarizes the three most frequently infested container types (with any immature stage) in each country per season. With the exception of India, where the most frequently infested container types in the dry and wet season were completely different.

All *Stegomyia* indices increased considerably in the wet season: The Container Index (CI = % of all water holding containers infested by *Aedes* larvae or pupae) increased by 78.3% (from 4.6 to 8.2), the Breteau Index (BI =  # containers infested per 100 houses) increased by 71.0% (from 22.4 to 38.3) and the House Index (HI = % of houses with ≧1 infested containers) increased by 56.3% (from 15.1 to 23.6). Likewise, the pupal production increased from the dry to the wet season by 31.2% (from 86 pupae per cluster to 130), while the pupae per person index PPI = # pupae per number of people) increased by 62.3% (from 0.162 pupae per person to 0.263). This increase in entomological indices in the wet season was observed across all sites even in the Philippines where they were storing water during the dry season (Table 2). The most productive container types (with close to or above 70% of total pupal production) are presented in Table 3.

**Table 3:** The most productive container types in the dry and wet season (private and public spaces combined) and how best to identify them

	Season	India	Indonesia	Myanmar	Philippines	Sri Lanka	Thailand
Number and type of productive containers^*^	Dry	Drum, cement tank, discarded containers	cement tank	Drum/ barrel, Cement tank, Spiritual flower bowl	Drum / barrel	Discarded containers, Tyre, drum/barrel	ceramic/earthern jar,Bucket
	Wet	Cement tank, Grinding stone, Drum/barrel	Cement tank, Drum/barrel; Flower vase	Spiritual flower bowl; cement tank; flower vases	Drum/barrels; Coconut, Ceramic jar	Bowl, tins/bottles	Bucket/bowls; tyres; tins/bottles
**Percentage of pupae produced by productive containers**	Dry	76	86.7	78.9	84	68.5	75
	Wet	70.6	62.1	69.8	76.5	78.1	65.7
**Number of productive container types missed if doing pupal survey in..**	Dry	1^**^	2	0	2	1	3
	Wet	0	0	0	0	1	1
**Number of productive container types missed (out of the important ones) if doing larveys in…**	Dry season	2/3^***^	0/1^****^	2/3	1/1	3/3	2/2
	Wet season	2/3	2/3	2/4	2/3	2/2	2/4

^*^Container type producing more than 70% of all pupae.

^**^Example : When doing the pupal survey during the dry season, one of four most productive container types in the wet season would not have been identified productivity surveys).

^****^However this productive container type identified by a larval survey was unimportant as only a small number of pupae (and larvae) was found during the dry season.

Regression analysis of pupal production at the household level showed that the factors associated with increased pupal production were the same in the dry and wet seasons: outdoor water containers, uncovered or partially covered, beneath shrubbery and not used for seven days or more (Table 4).

**Table 4:** Container characteristics significantly associated^a^ with the number of pupae per container identified in the multi-variate analysis of risk factors for dengue vector breeding during wet and dry season in private premises (outdoor and indoor)

	Wet season			Dry season		
Container	IRR^b^	95% CI	P-value	IRR^b^	95% CI	P-value
not under shrubbery	Reference			Reference		
fully or partially under shrubbery	0.51	0.33–0.78	0.002	0.50	0.25–0.98	0.045
used during past 7 days	Reference			Reference		
not used during past 7 days	6.74	4.37–10.37	<0.001	2.39	1.26–4.52	0.007
fully covered	Reference			Reference		
partially covered	3.79	1.53–9.34	0.004	7.95	2.53–24.92	<0.001
not covered	2.58	1.27–5.21	0.008	2.90	1.30–6.47	0.009

^a^ Results of negative binomial regression with clustering at the study cluster level.

^b^ Incidence rate ratio. Example: the expected pupal count for containers not used in the past 7 days is 6.74 times higher than that for containers used in the past 7 days.

#### Pupal productivity

**Figure 3:**
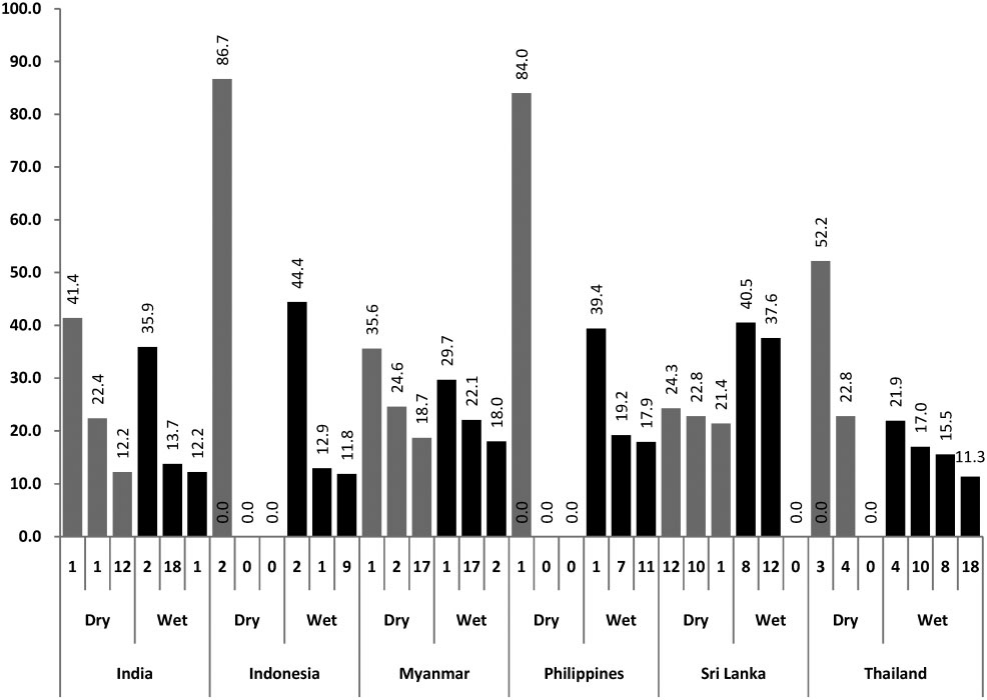
The containers producing the most pupae (% contribution to the total number of pupae; code of container type at the bottom of each column); Container codes: 1 drum/barrel; 2 cement tank; 3 ceramic/earthen jar; 4 bucket; 7 ceramic jar; 8 bowl; 9 flower vase; 10 tyre; 11 coconut shell; 12 discarded containers (tins, bottles etc); 17 spiritual flower bowl; 18 grinding stone.

The analysis of productive container types is presented in Table 3 and Figure 3. The container types most frequently infested by any immature stages (Figure 2) were different from the most productive container types (i.e. those which produce together more than 70% of all pupae) as shown in Figure 3. For example, the container types most frequently infested (with all immature stages) in the dry season in India were containers 10, 9 and 12 (tires, flower vases and discarded containers) and the three most productive container types in the dry season were 1, 2 and 12 (drums, cement tanks and discarded containers). Similar trends can be observed in all study sites. Table 3 shows how many productive container types would be missed if container types most frequently infested with all immature stages were used as proxies for pupal (and finally adult) densities. In Myanmar in the dry season, two out of three productive container types (for *Aedes* pupae) would be missed by conducting larval surveys alone. In Thailand, the Philippines and Sri Lanka, none of the productive container types for pupae would be identified by larval surveys.

##### Key productive container types during the dry and wet season

In two sites, only one container type produced a large proportion of pupae (86.7% in Indonesia and 84.0% in the Philippines) during the dry season; in Thailand two key container types produced 75.0% of pupae but in the other three sites, three key container types together were the most productive during the dry season. In the wet season the number of key productive container types increased: four sites had three key containers, each producing more than 70% of pupae, and India had four key container types. Only in Sri Lanka did the number of key productive container types decrease from three in the dry season to two in the wet season.

##### Optimal timing for determining key containers for Aedes production

When conducting the pupal productivity survey during the dry season, a variety of productive containers which appeared during the wet season were missed (Table 3). One key productive container type was missed in both India and Sri Lanka, two in Indonesia and Philippines and three in Thailand. Only in Myanmar the key container types remained the same in both seasons. In contrast, when doing the pupal productivity survey during the wet season, almost no key productive container types appearing in the dry season were missed in sites with higher pupal production (Myanmar, Indonesia, Philippines, India). In the sites with lower pupal production (Sri Lanka and Thailand), one productive container type in each would have been missed if the productivity survey had only occurred during the wet season.

## Disscussion

Although there were risk factors for vector breeding present both during the wet and dry season across study sites, social and environmental factors and vector control measures determined the variation of dengue vector breeding in the dry and wet season. In our study sites with mainly middle class neighborhoods and good-to-reasonable access to public services (Table 1), there were limited breeding opportunities for *Aedes* mosquitoes, which explains the relatively low entomological indices (Table 2). But also the large variation of productive container types (from grinding stones to large cement tanks) illustrates the social and environmental differences among our study areas. Additionally, the differences between the dry and wet seasons were striking. While increased rainfall, humidity and temperature favor vector breeding in the wet season,^24^–^26^ water storage may be enhanced in the dry season (as in the case of the Philippines in the present study). Nevertheless, pupal productivity was higher during the wet season, probably because our data demonstrated that the vectors preferred outdoor containers in shady places, filled with rainwater and that were rarely changed.

This study highlights the importance of determining ‘key productive container types’ which are responsible for the development of the majority of dengue vectors to their adult stage, as these were clearly different from containers infested with all *Aedes* immatures (represented by the classical *Stegomyia* indices). This distinction has been demonstrated previously in other parts of the world.^12^,^13^,^27^–^31^ The relationship between adult *Aedes* densities and pupal counts has been demonstrated by Focks and others.^11^,^12^,^30^ Linked to this is the concept of “targeted interventions,” whereby only the most pupal productive container types are targeted in vector control interventions, to maximize the effect on the potential adult vector population. This approach has been shown to be more cost-effective than routine measures which try to manage or treat all container habitats without targeting any specific container type.^10^ The findings of this study suggest that pupal productivity surveys would be best conducted during the wet season, given that the key container types identified during that survey included nearly all of those that were also of importance during the dry season. Pupal productivity surveys provide a vector surveillance tool for decision making for which container types to focus interventions and thus which kind of vector management to use, as this can depend on the container type.^6^,^11^,^33^ The calculation of pupal indices can also reflect dengue transmission risk. As an example: taking the overall PPI of 0.263 (average during the wet season, Table 2) in the average household with 4.5 inhabitants, there would be an average of 1.18 vectors per house (i.e., 0.263*4.5) or 0.59 female vectors. Considering the multiple biting habits of *Aedes* species and the dengue transmission threshold as calculated by Focks *et al.* (2000),^33^ the risk of dengue transmission is considerable particularly if the ambient air temperature is high (shortening the extrinsic incubation period) and herd immunity is moderate or low.

The traditional *Stegomyia* indices, which were widely applied during the *Ae. aegypti* eradication campaign in the Americas, are poor proxies for adult abundance but indicate the presence or absence of vectors^5^ and continue to serve for vector surveillance. Our study was able to address one of the practical questions often asked by dengue vector control services: What is the optimal timing for pupal productivity surveys to get representative information on the key productive container types? The study showed that doing the survey during the dry season would miss a number of productive containers during the wet season, but not the other way around; pupal productivity surveys during the wet season identified almost all productive container types relevant in both the dry and wet seasons. The different types of targeted interventions to be employed are explored in^10^ and will be further detailed in this special issue.
